# Healthcare Expenditures Associated With Comorbid Anxiety and Depression Among Adults With Migraine

**DOI:** 10.3389/fneur.2021.658697

**Published:** 2021-05-21

**Authors:** Monira Alwhaibi, Abdulkarim M. Meraya, Yazed AlRuthia

**Affiliations:** ^1^Department of Clinical Pharmacy, College of Pharmacy, King Saud University, Riyadh, Saudi Arabia; ^2^Department of Clinical Pharmacy, College of Pharmacy, Jazan University, Jizan, Saudi Arabia; ^3^Pharmacy Practice Research Unit, College of Pharmacy, Jazan University, Jizan, Saudi Arabia; ^4^Pharmacoeconomics Research Unit, College of Pharmacy, King Saud University, Riyadh, Saudi Arabia

**Keywords:** migraine, anxiety, depression, cost, healthcare

## Abstract

**Introduction:** Depression and anxiety are common among patients with migraine and usually associated with a humanistic and financial burden. This study aims to examine the direct healthcare expenditures among adults with migraine alone or with comorbid anxiety and/or depression.

**Methods:** This was a retrospective cross-sectional study using 2012, 2014, and 2016 Medical Expenditure Panel Survey data. Adult patients aged ≥22 years with migraine headache were included in the study. The direct healthcare expenditures of four migraine groups (migraine alone, migraine and anxiety, migraine and depression, and migraine and both conditions) were compared.

**Results:** There were 1,556 patients who met the inclusion criteria and eventually enrolled in the study. Approximately 42% of the study sample had migraine with comorbid depression and/or anxiety (16.1% have depression, 12.3% have anxiety disorder, and 13.9% have both). The mean total healthcare expenditures of adults with migraine alone ($6,461) were significantly lower than those with comorbid depression and anxiety ($11,102), comorbid anxiety ($10,817), and comorbid depression ($14,577). Migraine with comorbid anxiety and depression was significantly associated with incremental costs of $1,027 in outpatient and $662 emergency room healthcare expenditures and prescription drug compared to the migraine alone group.

**Conclusions:** The healthcare expenditures associated with migraine with comorbid depression and/or anxiety are significantly higher than those without mental health comorbidities. Therefore, regular depression and anxiety screening for patients with migraine may help reduce the healthcare expenditures associated with depression and/or anxiety comorbidities and improve the quality of care.

## Introduction

Migraine headache is a highly prevalent chronic condition among adults in the United States (US); around 16% of US adults suffer from migraine (1). The World Health Organization (WHO) ranked migraine headache as the third cause of disability measured in years lived with disability (2). Migraine is associated with poor health-related quality of life (3, 4), disability (4, 5), and missed working days (6). Migraine is also associated with medication overuse (7). Furthermore, it is associated with a great economic burden in terms of direct medical costs (8–10) and indirect costs due to absenteeism in the workplace (11). Individuals with migraine are more likely to have higher healthcare expenditures than those without migraine (8, 9). Individuals with migraine also have a higher healthcare services utilization rate, such as emergency room and physician office visits, compared to those without migraine (6, 8, 12).

Depression and/or anxiety are common among patients with migraine (13, 14). The likelihood of having a major depressive disorder along with migraine is significantly higher than their counterparts without migraine (15–17). Furthermore, it has been estimated that adults with migraine are two to four times more likely to suffer from depression and anxiety in comparison to the general population (3, 18–20). The comorbidity of depressive and anxiety disorders among migraine patients is associated with a high prevalence rate of disability (21), poor health-related quality of life (3, 4, 21), and a high rate of healthcare utilization and costs (22).

The economic burden of migraine is high, with an estimated annual cost of $17 billion in the US alone (23). However, this economic burden is significantly higher among patients with comorbid depression and/or anxiety (22, 24). Several studies have indicated that the healthcare expenditures associated with migraine with comorbid depression and/or anxiety can be double that of patients with migraine alone (22, 25). In a cohort study among adults with migraine using the 1-year data of a large employer-based insurance database, the presence of depression and/or anxiety along with migraine has resulted in higher direct medical costs than those with migraine alone ($5,589.5 migraine alone *vs*. $10,223.4 with anxiety and $10,582.2 with depression) (22). Moreover, in a cross-sectional study that included 2,400 subjects with migraine between 2006 and 2012, the mean annual total healthcare expenditures as well as migraine-related healthcare expenditures were significantly higher among migraine patients with depression compared to those without a comorbid depression ($10,012 *vs*. $4,740, *P* < 0.001, and $723 *vs*. $499, *P* = 0.014), respectively (25).

However, studies that compared the differences in healthcare expenditures among adults with migraine with or without comorbid anxiety and/or depression using nationally representative data are scarce. Thus, this study aimed to examine the differences in healthcare expenditures among adults with migraine alone and those with comorbid anxiety and/or depression, considering a myriad of factors that are likely to influence healthcare expenditures among this segment of the patient population. We hypothesized that adults with migraine alone have a lower healthcare expenditure as compared to those with comorbid anxiety and/or depression.

## Methods

### Study Design

This study employed a cross-sectional study design using the Medical Expenditure Panel Survey (MEPS) data of 2012, 2014, and 2016.

### Data Source

MEPS is a nationally representative complex panel survey design conducted by the Agency for Healthcare Research and Quality of the US non-institutionalized civilian population. It provides information on demographics, socioeconomic characteristics, medical conditions, health status, use of medical services, payments and charges, access to care, health insurance coverage, and other health-related data. The International Classification of Diseases, Ninth Revision, Clinical Modification (ICD-9-CM) codes was used to report the medical conditions. In 2016, the International Classification of Diseases, Tenth Revision, Clinical Modification (ICD-10-CM) codes were used to report medical conditions.

### Study Population

Adults, aged 22–64 years, with migraine headache (based upon ICD-9 code of “346,” ICD-10 code of “G43”) who were alive during the calendar years of 2012, 2014, and 2016 were included in this study. The study excluded adults without migraine headache and those aged <22 years. Three alternate years of data (2012, 2014, and 2016) were combined to increase the sample size. The use of alternate years is based on the recommendations of the Agency for Healthcare Research and Quality to avoid duplicate observations of the same participant (26).

### Measures

#### Dependent Variables

##### Type of and Total Healthcare Expenditures

The types of healthcare expenditures, such as inpatient, outpatient, prescription, emergency room, and other healthcare expenditures (e.g., dental, vision, and durable medical equipment use, and others), were included in the analysis. Furthermore, the total healthcare expenditures, which consisted of the sum of all types of healthcare expenditures (including inpatient, outpatient, prescription drugs, emergency visits, and other expenditures), were estimated. All healthcare expenditures were adjusted using the consumer price index and were expressed in 2016 constant dollars provided by the US Bureau of Labor Statistics (27).

#### Key Independent Variable

The primary independent variable was the presentation of migraine, which included four mutually exclusive groups (migraine alone, migraine and anxiety, migraine and depression, and migraine with both conditions). Anxiety and depression were identified using the following clinical diagnostic codes (anxiety: CCS code of “651,” ICD-10 code of “F40, F41;” depression: ICD-9 code of “296, 311,” ICD-10 code of “F34, F39, and F32”).

#### Other Independent Variables

The other independent variables that were considered were the sociodemographic characteristics, which included gender, age in years (22–39, 40–49, and 50–64), race/ethnicity (White, African American, Latino, and other), marital status (married, widowed, separated/divorced, and never married), education (less than high school, high school, and above high school), region of residence (Northeast, Midwest, South, and West), employment (unemployed, employed), health insurance (public, private, and uninsured), prescription medication insurance (insured, uninsured), and poverty status (poor: <100% federal poverty line; near poor: 100– <200%; middle income: 200– <400%; and high income: ≥400%). Other independent variables included personal health practices such as physical activity (three times/week, no exercise), smoking (smoker, non-smoker), perceived physical health (excellent/very good, good, and fair/ poor), and comorbid chronic health conditions such as diabetes, hypertension, and hyperlipidemia.

### Statistical Analysis

Descriptive statistics were used to describe the study sample across the four migraine groups. One-way analysis of variance (ANOVA) was used to compare the unadjusted means of the total healthcare expenditures across the four migraine groups. Generalized linear model (GLM) regressions with log link were used to estimate the healthcare expenditures associated with comorbid anxiety and depression among individuals with migraine after adjusting for the confounders (sociodemographic characteristics, health insurance status, personal health practices, perceived physical health, and comorbid chronic health conditions). The GLM is an attractive alternative to ordinary least squares regressions on log-transformed expenditures because it corrects for heteroscedasticity and avoids retransformation bias (28). We used GLM with log link and gamma family distribution to estimate the adjusted medical expenditures associated with comorbid anxiety and depression. We also used a two-part model to estimate inpatient expenditures as the majority of the adults in the sample had zero inpatient expenditures. The first part of the model estimates the probability of having zero expenditure *vs*. positive expenditures. The second part of the model uses GLM to estimate the expenditures conditional on having positive inpatient expenditures. A *p*-value <0.05 was considered statistically significant. The primary sampling unit, strata, and weights provided in the MEPS were all used in the analysis. All analyses were performed using survey procedures in the Statistical Analysis System, SAS 9.4 (SAS Institute Inc., Cary, NC, USA) and the Software for Statistics and Data Science (STATA 15.1).

## Results

### Description of the Study Sample

The characteristics of the study sample who met the inclusion criteria (*n* = 1,556) are shown in Table 1. The vast majority of patients were women (83.0%), were white (74.3%), and belong to the 22–39 years age group (40.3%). Approximately 16% of the study sample had depression, 12.3% had an anxiety disorder, and 13.9% had both conditions. Sociodemographic and economic factors, personal health practices, and coexisting chronic conditions were significantly different across the four migraine groups (*P* < 0.05). For example, the percentage of patients who reported employment among patients with migraine alone (64.2%) is significantly higher than their counterparts from other migraine groups, which were 14.3, 11.6, and 9.9% among patients with comorbid depression, comorbid anxiety, and comorbid depression and anxiety, respectively.

**Table 1:** Characteristics of the study sample and number and row percentage of characteristics by migraine group among adults with migraine (Medical Expenditure Panel Survey data of 2012, 2014, and 2016).

	**Total sample**		**Migraine alone**		**Migraine and depression**		**Migraine and anxiety**		**Migraine and depression and anxiety**		
	***N***	**Wt.%**	***N***	**Wt.%**	***N***	**Wt.%**	***N***	**Wt.%**	***N***	**Wt.%**	***P*-value**
All	1,556	100	912	57.7	243	16.1	185	12.3	216	13.9	
Age in years											
22–39	661	40.3	419	62.0	79	13.1	90	13.6	73	11.4	0.049
40–49	421	26.9	240	57.7	70	16.9	43	9.9	68	15.5	
50–64	474	32.8	253	52.5	94	19.1	52	12.6	75	15.7	
Gender											
Women	1,307	83.0	743	56.2	205	15.9	167	13	192	15	0.090
Men	249	17.0	169	65.2	38	17.0	18	8.9	24	8.8	
Race/ethnicity	
White	850	74.3	458	55.3	141	16.8	116	12.7	135	15.2	0.020
African American	251	8.6	168	65.6	43	18.6	22	8.5	18	7.3	
Latino	357	12.1	221	63	47	12.4	36	11.7	53	12.9	
Others	98	5.0	65	68.2	12	9.1	11	13.2	10	9.4	
Marital status											
Married	782	56.8	505	62.4	105	14.7	80	10.0	92	12.9	<0.0001
Widow	229	13.1	106	40.3	49	21.2	30	15.7	44	22.8	
Sep/Div	387	21.2	204	52.3	61	17.1	58	17.2	64	13.5	
Never married	158	8.9	97	66.7	28	15.2	17	9.8	16	8.4	
Education level											
LT HS	142	5.8	77	50.2	37	28.1	11	7.7	17	14.0	0.076
HS	198	11.8	110	58	34	16.4	23	11.5	31	14	
>HS	922	64.3	531	56.7	127	14.8	117	13	147	15.6	
Missing	294	18.1	194	63.7	45	16.7	34	11.7	21	7.9	
Region											
Northeast	268	17.6	156	58.9	34	11.6	38	14.3	40	15.1	0.670
Mid-west	367	25.0	199	53.9	70	18.8	41	12	57	15.3	
South	504	32.9	307	59.8	76	17.1	55	10.5	66	12.6	
West	417	24.5	250	58.0	63	15.1	51	13.3	53	13.5	
Employment											
Employed	1,061	73.9	708	64.2	134	14.3	125	11.6	94	9.9	<0.0001
Not employed	495	26.1	204	39.4	109	21	60	14.3	122	25.3	
Poverty status^a^											
Poor	327	14.2	141	38.6	69	20.9	43	15.9	74	24.6	<0.0001
Near poor	317	15.1	184	53.1	48	15.8	38	14.6	47	16.5	
Middle income	440	27.8	273	57.2	59	16.8	53	12.1	55	13.9	
High income	472	42.9	314	66.1	67	14.1	51	10.3	40	9.5	
Health insurance											
Private	1,002	75.0	643	62.6	138	15.0	121	11.8	100	10.6	<0.0001
Public	416	18.1	182	38.9	85	20.2	55	16.2	94	24.8	
Uninsured	138	6.8	87	54.5	20	17.2	9	6.8	22	21.5	
Rx insurance											
Rx insurance	884	68.1	569	62.8	115	14.5	114	12.2	86	10.6	<0.0001
No Rx insurance	672	31.9	343	47.0	128	19.5	71	12.3	130	21.1	
General health											
Excellent/very good	604	43.0	454	75.2	60	10.7	54	8.2	36	6.0	<0.0001
Good	519	32.2	298	53.4	89	17.9	66	14.6	66	14.1	
Fair/poor	433	24.8	160	33.2	94	23	65	16.3	114	27.5	
Physical activity											
3/week	657	42.9	439	65.1	78	12.6	77	13.2	63	9.1	<0.0001
No exercise	895	56.8	471	52.3	165	18.8	107	11.6	152	17.4	
Smoking											
Current smoker	271	16.3	121	41.7	54	20.4	37	13	59	24.9	<0.0001
Others	1,189	76.9	731	60.8	173	15.2	140	12.4	145	11.5	
Missing	96	6.8	60	61.4	16	15.2	8	8.8	12	14.6	
Heart											
Yes	154	8.9	62	35.9	31	21.8	16	12.6	45	29.7	<0.0001
No	1,402	91.1	850	59.9	212	15.5	169	12.2	171	12.4	
Hypertension											
Yes	393	24.0	178	45.7	82	19.7	54	15.2	79	19.5	0.001
No	1,163	76.0	734	61.5	161	14.9	131	11.3	137	12.2	
Diabetes											
Yes	128	7.2	54	39.9	21	13.9	19	17.7	34	28.5	<0.0001
No	1,428	92.8	858	59.1	222	16.2	166	11.8	182	12.8	
Hyperlipidemia											
Yes	781	48.3	433	55.7	137	17.2	88	10.7	123	16.5	0.091
No	775	51.7	479	59.6	106	15	97	13.8	93	11.6	
Asthma											
Yes	240	14.8	108	45.0	48	19.6	30	14.9	54	20.5	0.006
No	1,316	85.2	804	60.0	195	15.5	155	11.8	162	12.8	
COPD											
Yes	255	16.3	105	42.1	55	21.8	33	12.8	62	23.4	<0.0001
No	1,301	83.7	807	60.8	188	15	152	12.2	154	12.1	
Arthritis											
Yes	540	34.8	218	39.4	120	24.3	84	15.5	118	20.8	<0.0001
No	1,016	65.2	694	67.5	123	11.7	101	10.5	98	10.3	
GERD											
Yes	204	13.5	84	38.4	49	24.8	20	12.2	51	24.6	<0.0001
No	1,352	86.5	828	60.8	194	14.7	165	12.3	165	12.3	
Cancer											
Yes	96	7.2	35	36.0	23	26.8	17	22.0	21	15.2	<0.0001
No	1,460	92.8	877	59.4	220	15.2	168	11.5	195	13.8	

*Based on 1,556 adults with migraine headache, aged≥22 years, and who were alive during the calendar years 2012, 2014, and 2016. Missing indicators for education and smoking were used but not presented in the table*.

a *Poor, <100% federal poverty line; near poor, 100 to <200%; middle income, 200 to <400%; high income, >400%*.

*COPD, chronic obstructive pulmonary disease; GERD, gastroesophageal reflux disease; HS, high school; LT, less than; Rx, prescription drug; N, number; Wt, weighted; Sep/Div, separated/divorced*.

### Healthcare Expenditures by Migraine Groups

The differences in healthcare expenditures associated with migraine between adults with migraine alone and those with comorbid anxiety and/or depression are shown in Table 2. The mean total healthcare expenditures among patients with migraine alone were significantly lower than their counterparts with comorbid anxiety and/or depression. Similarly, the mean inpatient, outpatient, prescription, and emergency healthcare expenditures were significantly lower among patients with migraine alone compared to other migraine groups. For example, total healthcare expenditures for individuals with migraine alone were $6,461, which were significantly lower than the total healthcare expenditures for individuals with migraine and either comorbid anxiety ($11,102) or depression ($10,817) or with both comorbid anxiety and depression ($14,577).

**Table 2:** Mean healthcare expenditures by migraine groups among adults with migraine (Medical Expenditure Panel Survey data of 2012, 2014, and 2016).

	**Migraine alone**		**Migraine and depression**		**Migraine and anxiety**		**Migraine and depression and anxiety**		
	**Mean ($)**	**SE**	**Mean ($)**	**SE**	**Mean ($)**	**SE**	**Mean ($)**	**SE**	***p*-value**
Expenditures									
Total	6,461	634	11,102	1,446	10,817	1,382	14,577	2,020	<0.0001
Inpatient	1,391	353	1,250	421	1,845	592	3,206	957	0.002
Outpatient	2,360	207	4,836	1,216	4,374	781	4,723	404	<0.0001
Prescription	1,619	224	3,922	542	3,234	557	4,518	455	<0.0001
Emergency room	439	66	535	192	578	122	1,419	724	0.036
Other	652	86	560	83	785	299	710	111	0.552

*Based on 1,556 adults with migraine headache, aged 22 years and older, and who were alive during the calendar years 2012, 2014, and 2016. p-value represents significant mean differences by migraine groups using ANOVA. Other expenditures included dental, vision, durable medical equipment use, and others*.

*$, US dollar; SE, standard error*.

### Adjusted Healthcare Expenditures by Migraine Groups

The adjusted differences in healthcare expenditures between patients with migraine alone and those with comorbid anxiety and/or depression are shown in Table 3. Migraine with comorbid anxiety and depression was significantly associated with incremental costs of $1,027 in outpatient and $662 emergency room healthcare expenditures, compared to the migraine alone group after controlling for a multitude of covariates. Besides this, migraine with comorbid anxiety and/or depression was significantly associated with incremental costs of the prescription drug, compared to the migraine alone group after controlling for a multitude of covariates. However, migraine with comorbid depression and/or anxiety was not associated with significant incremental inpatient or other healthcare costs. Moreover, the incremental total healthcare costs were not significant for migraine patients with comorbid depression and/or anxiety.

**Table 3:** Intercept and parameter estimates for the migraine groups from generalized linear models with log link and gamma distribution on healthcare expenditures among adults with migraine (Medical Expenditure Panel Survey 2012, 2014, and 2016).

		**Migraine and depression**				**Migraine and anxiety**				**Migraine and depression and anxiety**			
	**Intercept (SE)**	**β**	**SE**	**Incremental cost**	***p*-value**	**β**	**SE**	**Incremental cost**	***p*-value**	**β**	**SE**	**Incremental cost**	***p*-value**
**Unadjusted generalized linear model with log link and gamma distribution**													
Total	8.77 (0.09)	0.54	0.15	$4,641	0.002	0.51	0.16	$4,356	0.005	0.81	0.17	$8,116	<0.0001
Inpatient	9.78 (0.78)	−0.28	0.42	–$141	0.799	0.04	0.33	$454	0.525	−0.07	0.30	$1,815	0.086
Outpatient	7.76 (0.08)	0.71	0.21	$2,476	0.026	0.62	0.20	$2,014	0.014	0.69	0.12	$2,363	<0.0001
Prescription	7.38 (0.14)	0.88	0.19	$2,302	<0.0001	0.69	0.23	$1,616	0.009	1.02	0.17	$2,899	<0.0001
Emergency	6.08 (0.15)	0.27	0.27	$95	0.639	0.27	0.27	$140	0.331	1.17	0.52	$979	0.177
Other	6.47 (0.13)	−0.15	0.19	–$91	0.432	0.18	0.40	$133	0.668	0.08	0.22	$58	0.710
**Adjusted generalized linear model with log link and gamma distribution**													
Total	10.61 (0.41)	0.11	0.11	1,025	0.330	0.09	0.12	$871	0.444	0.14	0.14	$1,295	0.312
Inpatient	10.8 (0.68)	−0.48	0.27	–$1,332	0.029	−0.1	0.34	–$707	0.326	−0.23	0.29	–$589	0.453
Outpatient	8.46 (0.43)	0.15	0.12	$515	0.224	0.23	0.14	$816	0.129	0.29	0.13	$1,027	0.041
Prescription	8.75 (0.46)	0.43	0.14	$1,296	0.005	0.52	0.16	$1,598	0.006	0.39	0.16	$1,128	0.017
Emergency	7.89 (0.92)	0.37	0.29	$213	0.281	0.68	0.36	$460	0.139	0.88	0.31	$662	0.032
Other	6.11 (0.58)	−0.16	0.16	–$109	0.307	−0.32	0.18	–$197	0.075	−0.04	0.21	–$28	0.849

*Based on 1,556 adults with migraine headache, aged 22 years and older, and who were alive during the calendar years 2012, 2014, and 2016. p-values denote statistical significance in parameter estimates from generalized linear models on expenditures with log link and gamma distribution. Other expenditures included dental, vision, durable medical equipment use, and others*.

*Reference group for migraine groups = migraine alone.*

*$, US dollar; B, coefficient; p-value, probability value; SE, standard error*.

## Discussion

The present study compared healthcare expenditures among subjects with migraine when diagnosed alone or with comorbid anxiety and/or depression. The study findings indicate that around half of adults with migraine had anxiety and/or depression; this is consistent with the findings of previously published studies (21). The high prevalence of depression and anxiety among adults with migraine and the significantly higher healthcare-related costs associated with it compared to the general population emphasize the need for incorporating depression and anxiety disorders screening into the routine clinical practice of managing migraine among adults. Adults with migraine who were diagnosed with comorbid anxiety and/or depression incurred two to three times higher total healthcare expenditures compared to those with migraine alone. This is consistent with the study of Pesa et al. who found that the presence of depression and/or anxiety along with migraine resulted in significantly higher total direct medical costs ($5,589 migraine alone *vs*. $10,223 with anxiety and $10,582 with depression) (22). Furthermore, Wu *et al*. reported a higher mean annual total health expense for those with depression ($4,740 migraine alone *vs*. $10,012 with depression, *P* < 0.001) (25).

What distinguishes the current investigation from the previously published studies is the fact that the healthcare expenditures for anxiety and anxiety with depression comorbidity were identified after adjusting for a myriad of variables (22, 25). Significant differences between migraine groups with respect to outpatient healthcare expenditures and prescription drugs only were found. Migraine with comorbid anxiety and depression was significantly associated with incremental costs of $1,027 in outpatient and $662 emergency room healthcare expenditures and $1,128 in prescription drug compared to the migraine alone group. The positive association between comorbid anxiety and depression and healthcare expenditures among adults with migraine has many potential explanations. For example, depression and/or anxiety may exacerbate migraine symptoms and increase the healthcare utilization rate, resulting in higher overall healthcare expenditures. Multiple observational studies with nationally representative samples among adult patients with migraine have reported that the comorbidity of migraine and depression was associated with higher rates of emergency department visits and all-cause office visits compared to those without depression (20, 25). Besides this, it is well-established that the comorbidity of migraine with depression and/or anxiety is associated with a higher rate of prescription drug utilization as reported by SMILE that was conducted among migraine patients visiting primary care clinics in France (29). Effective management of depressive and anxiety disorder among patients with migraine may reduce the rate of healthcare utilization and expenditures and improve the patients' quality of life. Therefore, future studies should examine the economic impact of proper depression and/or anxiety management among patients with migraine and whether this will lower the overall healthcare expenditures.

However, it has to be noted that migraine with comorbid depression and/or anxiety was associated with an increase in the total healthcare expenditures and inpatient and other expenditures, resulting in higher total healthcare expenditures compared to the migraine alone group. This association did not reach a statistically significant level in the adjusted analysis, where no significant difference in total healthcare expenditures, inpatient expenditures, and other expenditures was observed between the migraine groups. The lack of significant differences in the total healthcare expenditure between groups could be attributed to the fact that these psychiatric conditions can be associated with migraine at the sub-clinical level (i.e., sub-threshold level), which means that the “migraine alone” group could be an inadequate control group (30, 31). One unexpected finding was the lower inpatient costs among patients with depression comorbidity, although depression comorbidity is associated with a higher rate of hospitalization and emergency room visits compared to migraine patients without depression (20, 25). These unexpected findings warrant further examination in future studies.

Several practical implications can emanate from the current study. The higher outpatient expenditures among migraine patients with comorbid depression and/or anxiety highlight the need to improve the quality of care provided to this segment of the patient population to improve their adherence to prescribed drug regimens which will eventually lower the rate of emergency room visits and hospitalization, resulting in lower overall healthcare costs. Besides this, regular depression and anxiety screening for patients with migraine may also reduce the healthcare expenditures associated with migraine and improve the quality of care through the early detection and management of these conditions.

Although this study has multiple merits, such as the use of nationally representative data, the control for multiple medical and socioeconomic factors, and the robustness of the findings using different statistical models, it should be interpreted in the context of some limitations. These limitations, such as the lack of control for the severity and the duration of the illness, should be acknowledged. This study did not also adjust the cost based on the frequency of migraine attacks in the four groups compared due to the lack of this data. Besides this, the over-the-counter expenditures are not captured in the MEPS database. This study has not also compared the healthcare expenditure with time. There is also lack of data about the subtype of migraine (episodic, chronic, with aura), preventive treatment, and medication overuse. Besides this, only direct costs from all causes are reported, as the MEPS data does not provide specific costs of the subgroup of migraine.

## Conclusions

The current study has provided an updated estimate of the healthcare expenditures associated with migraine management with or without comorbid depression and/or anxiety among the adult US population. Its findings suggest that healthcare providers should be aware of the high prevalence of anxiety and depression among migraine patients that necessitates early detection and management of these comorbid conditions alongside migraine given the higher outpatient healthcare expenditures associated with these comorbid conditions.

## Data Availability Statement

The datasets generated for this study can be found in online repositories. The names of the repository/repositories and accession number(s) can be found below: The dataset supporting the conclusions of this article is available from the Medical Expenditure Panel Survey (MEPS) database and openly made available for researchers at the following website: https://meps.ahrq.gov/data_stats/download_data_files.jsp.

## Author Contributions

MA, YA, and AM have all contributed to the study conception and design. Data analysis was performed by MA and AM. The first draft of the manuscript was written by MA, YA, and AM. The authors have all commented on previous versions of the manuscript and read and approved the final manuscript.

## Conflict of Interest

The authors declare that the research was conducted in the absence of any commercial or financial relationships that could be construed as a potential conflict of interest.

## Abbreviations

WHO: World Health Organization
YLDs: years lived with disability
HRQoL: health-related quality of life
MEPS: Medical Expenditure Panel Survey
AHRQ: Agency for Healthcare Research and Quality
ICD-9-CM: The International Classification of Diseases, Ninth Revision, Clinical Modification
ANOVA: analysis of variance
GLM: generalized linear model
SAS: statistical analysis system
STATA: Software for Statistics and Data Science.
